# Transcriptome analysis of extant cotton progenitors revealed tetraploidization and identified genome-specific single nucleotide polymorphism in diploid and allotetraploid cotton

**DOI:** 10.1186/1756-0500-7-493

**Published:** 2014-08-06

**Authors:** Xueying Guan, Gyoungju Nah, Qingxin Song, Joshua A Udall, David M Stelly, Z Jeffrey Chen

**Affiliations:** Institute for Cellular and Molecular Biology and Center for Computational Biology and Bioinformatics, The University of Texas at Austin, Austin, Texas 78712 USA; Department of Plant and Wildlife Sciences, Brigham Young University, Provo, Utah 84602 USA; Department of Soil and Crop Sciences, Texas A&M University, College Station, TX 77843 USA

**Keywords:** Cotton, Polyploidy, *G. hirsutum*, *G. arboreum*, *G. raimondii*, EST, GNPs, mRNA sequencing, Transcriptome

## Abstract

**Background:**

The most widely cultivated cotton (*Gossypium hirsutum* L., AD-genome) is derived from tetraploidization between A- and D-genome species. *G. arboreum* L. (A-genome) and *G. raimondii* Ulbr. (D-genome) are two of closely-related extant progenitors. Gene expression studies in allotetraploid cotton are complicated by the homoeologous loci of A- and D-genome origins. To develop genomic resources for gene expression and cotton breeding, we sequenced and assembled expressed sequence tags (ESTs) derived from *G. arboreum* and *G. raimondii*.

**Results:**

Roche/454 FLX sequencing technology was employed to sequence normalized cDNA libraries prepared from leaves, roots, bolls, ovules, and fibers in *G. arboreum* and *G. raimondii*, respectively. Sequencing reads from two independent libraries in each species were combined to assemble high-quality EST contigs. The combined sequencing reads included 1,699,776 from A-genome and 1,464,815 from D-genome, which were clustered into 89,588 contigs in the A-genome and 65,542 contigs in the D-genome. These contigs represented ~80% of EST collections in Cotton Gene Index 11 (CGI11, March 2011). Compared to the D-genome transcript database, 27,537 and 10,452 contigs were unique transcripts in A and D genomes, respectively. Further analysis using self-blastn reduced the unigene contig number by 52% in A-genome and 57% in D-genome, suggesting that 50% or more of contigs are paralogs or isoforms within each species. The majority of EST contigs (73–81%) were conserved between A- and D-genomes, whereas 27% and 19% contigs were specific to A- and D-genomes, respectively. Using these ESTs, we generated a total of 75,754 genome-specific single nucleotide polymorphism (SNP) (gSNPs or GNPs) or homoeologous-specific SNPs (hSNPs) of 10,885 contigs or genes between A and D genomes, indicating a possibility of separating allelic expression for those genes in allotetraploid cotton.

**Conclusions:**

Expressed genes are highly redundant within each diploid progenitor and between A and D progenitor species, suggesting that diploid progenitors in cotton are likely ancient tetraploids. This large set of A- and D-genome ESTs and GNPs will be valuable resources for genome annotation, gene expression, and crop improvement in allotetraploid cotton.

**Electronic supplementary material:**

The online version of this article (doi:10.1186/1756-0500-7-493) contains supplementary material, which is available to authorized users.

## Background

Cotton is the most widely used source of natural and renewal fiber. The trading value of cotton has been ~8 billion annually in the past decades, which ranks cotton among top five crops in the US (http://www.epa.gov/agriculture/ag101/cropmajor.html). The modern cultivated cotton is dominated by two allotetraploid species, the Upland cotton (*Gossypium hirsutum* L.) and the Sea Island cotton (*G. barbadense* L.) [1]. These allotetraploid species were formed by hybridization and polyploidization 1–2 million years ago (Mya), followed by domestication and selection of fiber traits [2]. Although the exact progenitors of modern allotetraploid cotton are unknown, extant species of diploid progenitors include *G. herbaceum* L. (A-genome) and *G. arboreum* L. (A-genome) originating in the old world (Africa and Asia) and *G. raimondii* L. (D-genome) originating in the new world (America).

The size of the A-genome is estimated to be ~1.8 Gigabases (Gb) [3], which is almost twice as the size of the D-genome (0.98 Gb) [3, 4]. The size variation between A and D genomes is associated with the long-terminal-repeat retrotransposons (LTRs) [5, 6]. Indeed, D-genome comprises 61% of LTRs, which is the least amount of repetitive DNA among eight *Gossypium* species examined [5]. The D-genome has been recently sequenced, revealing an evolutionary process of cotton fiber domestication [7]. Although D-genome cotton species produce very short fiber with poor quality, in allotetraploid cotton more quantitative trait loci (QTLs) of the fiber quality are associated with the D-genome than with the A-genome [8, 9]. However, more genes encoding cell-fate regulatory factors and cell-cycle control components are expressed in the A-genome than in the D-genome [10, 11], and some fiber QTLs are more associated with the A-genome than the D-genome [12, 13]. Both genomes may also contribute equally to superior fiber agronomic traits associated with polyploidization and domestication in allotetraploid cotton [7, 14].

In allotetraploid cotton, expression of duplicated genes is biased towards A- or D-genome [15–17]. These results are based on microarray platforms using annotation of current ESTs derived mainly from allotetraploid cotton (*G. hirsutum*), which could contain some mixed sequences within each presumed homoeologous locus because it is difficult to discriminate sequence reads of A- and D-genome origins in allotetraploid species. To date, the largest cotton cDNA collection is Gossypium unigene v1.0 (http://www.cottongen.org/node/49448), which contains conservative sequences from several cotton species, including *G. hirsutum*, *G. barbedense*, *G. herbaceum*, *G. darwinii. G. mustelinum*, *G. arboreum*, and *G. tomentosum*, but limited numbers of ESTs from diploid progenitors [18].

In this study, we employed Roche/454 FLX System to perform mRNA sequencing using normalized cDNA libraries prepared from leaves, roots, stems, ovules, and fibers in *G. arboreum* L. (A-genome) and *G. raimondii* (D-genome). Two independent sequencing libraries were made to obtain a large number of ESTs in these species. These ESTs represent ~80% of all EST collections in cotton databases. Using the ESTs, we investigated expressed genes and gene duplication within each species and between these two species and developed a comprehensive list of GNPs that are useful for discriminating locus or allele-specific expression patterns in allotetraploid cotton.

## Results and discussion

### Sequencing and assembly of A- and D-genome ESTs

Sequence libraries were made from mRNA prepared from multiple tissues including young leaves, roots, stems, ovules, and fibers in *G. arboreum* and young leaves, stem and whole flowers in *G. raimondii*. To increase the abundance of poorly expressed genes, mRNA libraries were normalized using the procedure involving kamchatka crab duplex-specific nucleases (Evrogen and Axxora, LLC, Farmingdale, NY) [19]. To increase the sequencing coverage and depth, two independent mRNA libraries were constructed and sequenced. The first set of 454 Titanium reads was assembled at The University of Texas at Austin and included a total of 1,699,776 reads from the A-genome, and 1,464,815 reads from the D-genome. The assembly of these reads resulted in 62,609 contigs with an average contig size (N50) of 1,032-bp for A-genome and 34,908 contigs (N50 of 1,107-bp) for D genome. The second assembly included 89,185 contigs (N50 of 628.6-bp) from the A genome and 68,984 contigs (N50 of 675.6-bp) from the D genome at Brigham Young University [17].

**Figure 1:**
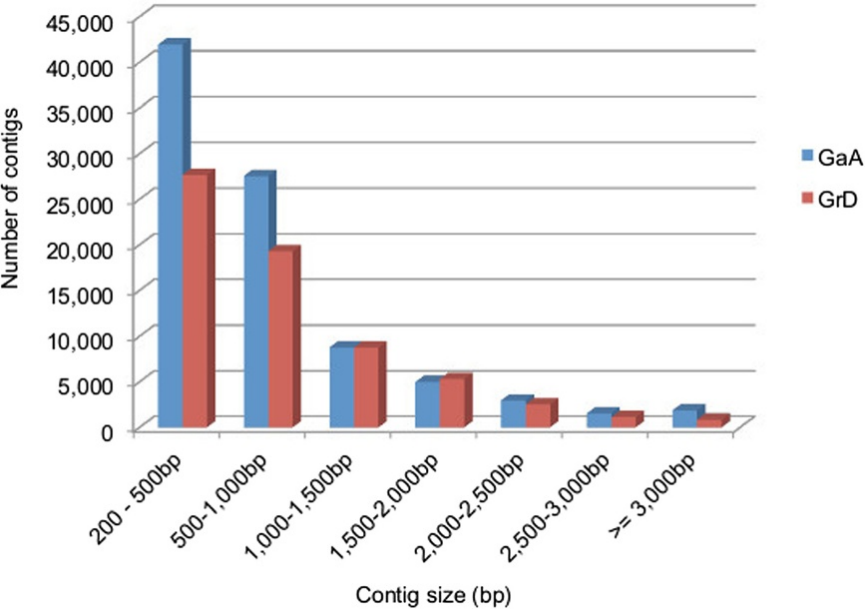
**Size distribution of GaA and GrD EST assemblies.**

To improve assembly quality, we filtered out contigs shorter than 200-bp because of the poor base quality and short size and merged two sets of EST assemblies. As a result, the contig number greater than 500-bp was increased (1.7-fold) from 27,574 to 47,670 for the A genome and from 21,763 to 37,869 for the D genome. Likewise, the average size greater than 500-bp increased from 940.7 bp to 1,184.5 bp (243.8-bp increase) for A-genome and from 994.5 bp to 1,198.4 bp (203.9-bp increase) for D-genome. The number of contigs remained similar for both A- (from 40,726 to 47,670) and D- (from 35,197 to 37,869) genomes. The merged ESTs were assembled into 89,588 contigs for the A-genome (GaA, N50 of 805.9-bp) and 65,542 contigs for the D-genome (GrD, N50 of 839.6-bp). Over 80% of A (88%) and D (84%) contigs ranged from 200 to 1,500-bp (Figure 1 and Additional file 1: Table S1). The average length of ESTs for GaA and GrD is longer than those of previously published ESTs in allotetraploid cotton (763 bp) [11].

**Figure 2:**
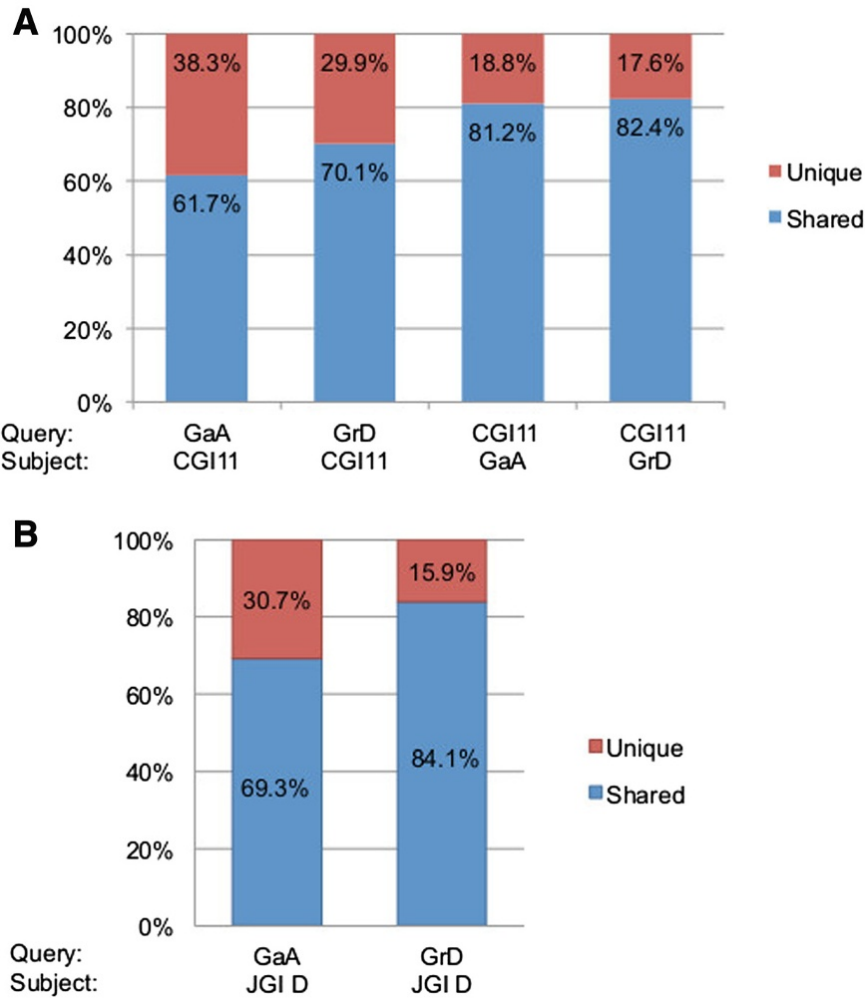
**Comparison revealed the coverage and novelty of GaA and GrD EST datasets.** BLASTN was used to analyze the representation of ESTs in cotton EST datasets using reciprocal combinations of the “query” and the “subject” between **(A)** CGI11, GaA and GrD ESTs, respectively and **(B)** JGI-D, GaA and GrD ESTs, respectively. An e-value of (<e^-10^) was used as the criteria for the common genes shared between EST libraries. JGI-D, and GaA and GrD ESTs, respectively. An e-value of (<e^-10^) was used as the criteria to identify the common genes shared in EST libraries. An e-value of (<e^-10^) was used to select the common set of genes shared between each other comparison.

To estimate the transcriptome coverage and novelty, we compared GaA and GrD ESTs with those in CGI11 that comprised 117,992 EST contigs in cotton varieties of diploid and allotetraploid species using reciprocal BLASTN with an e-value score below e^-10^ (Figure 2A). Using CGI11 as the query, the majority of contigs in CGI11 matched GaA (81.2%) and GrD (82.4%), respectively, suggesting a good representation of GaA and GrD ESTs in the current EST collections. Using the reverse query, GaA and GrD ESTs matched 61.7% and 70.1% of contigs, respectively in CGI11, indicating 38.3% of new transcripts in GaA and 29.9% in GrD, respectively. This is probably because of increased sequencing depth in two independent libraries, as well as the normalization of mRNA libraries to include the poorly expressed genes. These GaA and GrD EST assemblies provide unique and additional useful resources for genomic studies.

GaA and GrD EST assemblies were searched against annotated transcripts based on DOE Joint Genome Institute: Cotton D V2.0 (JGI-D) (http://www.phytozome.net/cotton.php#C3) [7]. With the e-value cut-off of 1e-10, we identified unique ESTs present in GaA and GrD EST assemblies. Compared to the JGI-D annotation, 27,537 contigs (30.7%) were unique to A-genome, and 10,452 contigs (15.9%) were unique to D-genome (Figure 2B). These unique ESTs are the genes that are expressed in other tissues such as roots and shoots, poorly expressed genes, non-coding RNAs and/or gene fragments in the D-genome. These new ESTs provide additional transcriptional information for cotton genome annotation.

### Characterization of A and D transcriptomes

**Figure 3:**
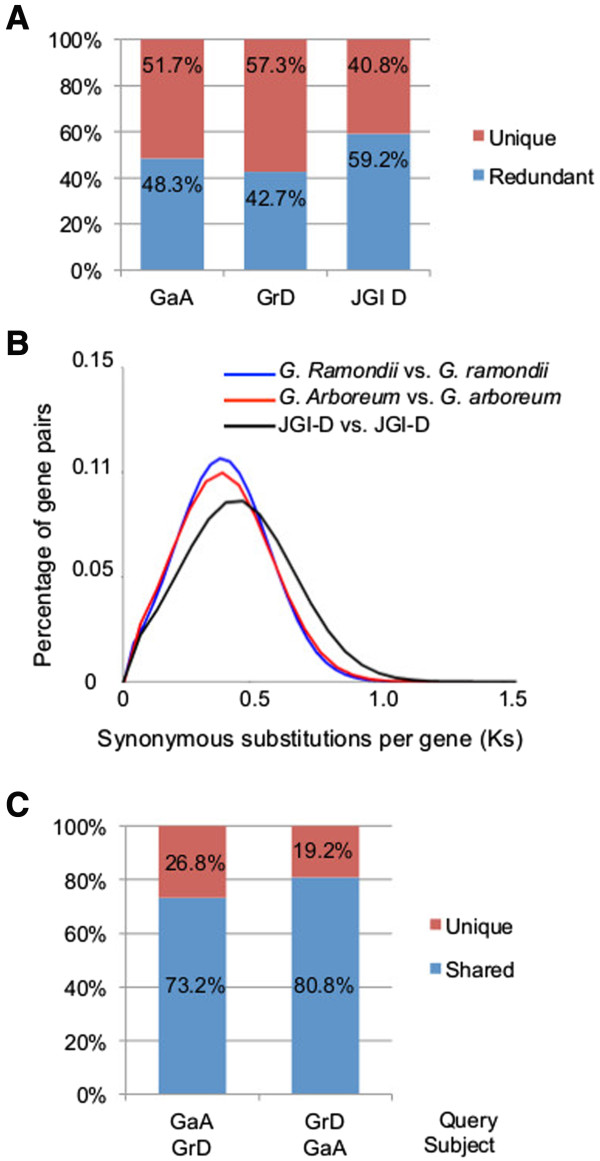
**Estimation of variance of two cotton diploid transcriptomes. (A)** Redundancy of ESTs in A- and D-genome species. Using self-BLASTN analysis (e-value < e^-100^), redundant ESTs (either isoforms or paralogs) were identified. **(B)** Frequency distribution of Ks for orthologous and paralogous gene pairs between GaA, GrD, and JGI-D sequences. **(C)** Specificity of cotton A and D transcriptomes. Reciprocal BLASTN analyses were performed between GaA and GrD assemblies. The cut-off e-value for uniqune and shared genes was e^-10^.

Most plants including cotton underwent one or more rounds of whole genome duplication or polyploidization [20–22]. To estimate duplicated transcripts in the A- and D-genome species, we performed self-BLASTN for GaA and GrD with e-value less than e^-100^ (Figure 3A). Compared to the total contigs, 48.3% and 42.7% of GaA and GrD ESTs and contigs were multiple-copy, respectively, and numbers of single-copy contigs were 43,302 for GaA and 28,019 for GrD (Figure 3A). The results remained similar using the annotated transcripts in the JGI-D in the place of GrD (Figure 3A). The JGI-D contained 37,505 annotated genes and 77,267 protein-coding transcripts [7].

Multiple genome duplication events occurred during cotton evolution [7, 23]. Evolutionary analysis indicated that in the D-genome cotton (*G. ramondii*) whole genome duplication (WGD) occurred after split from the closest genome *Theobroma cacao* [7]. This polyploidy event was estimated to be 13–20 Mya. As a result, 2,355 duplicate blocks were present across the D-genome, and ~40% of paralogous genes were present in more than one block [24]. We used the rate of nonsynonymous substitution per gene (Ks) to determine WGD events. The value of the highest peak (Ks = 0.4) of the Ks distributions of orthologs and paralogs between *G. ramondii* itself and *G. arboreum* and *G. ramondii* were very similar (Figure 3B). These data may imply that WGD in A- and D-genomes occurred at a similar timeframe. The WGD could increase the rate of evolution for these related species. Our analysis confirmed expression of these duplicate genes from WGD, which are preserved after polyploidization.

We next tested divergence between transcriptomes of A- and D-genomes that were predicted to diverge 7–10 Mya [25]. Using reciprocal BLASTN searches between GaA and GrD ESTs, we found that 73.2% (65,591) of GaA contigs and 80.8% (52,922) of GrD contigs were shared (Figure 3C). This suggests that the majority of transcriptomes was conserved after divergence between A- and D-genome species. The unique genes between these two species could be related to species-specific gene loss or gain and tissue-specific expression of genes and/or proportion of genes that are important to fiber development because these two species have very different fiber morphologies and properties [26].

**Figure 4:**
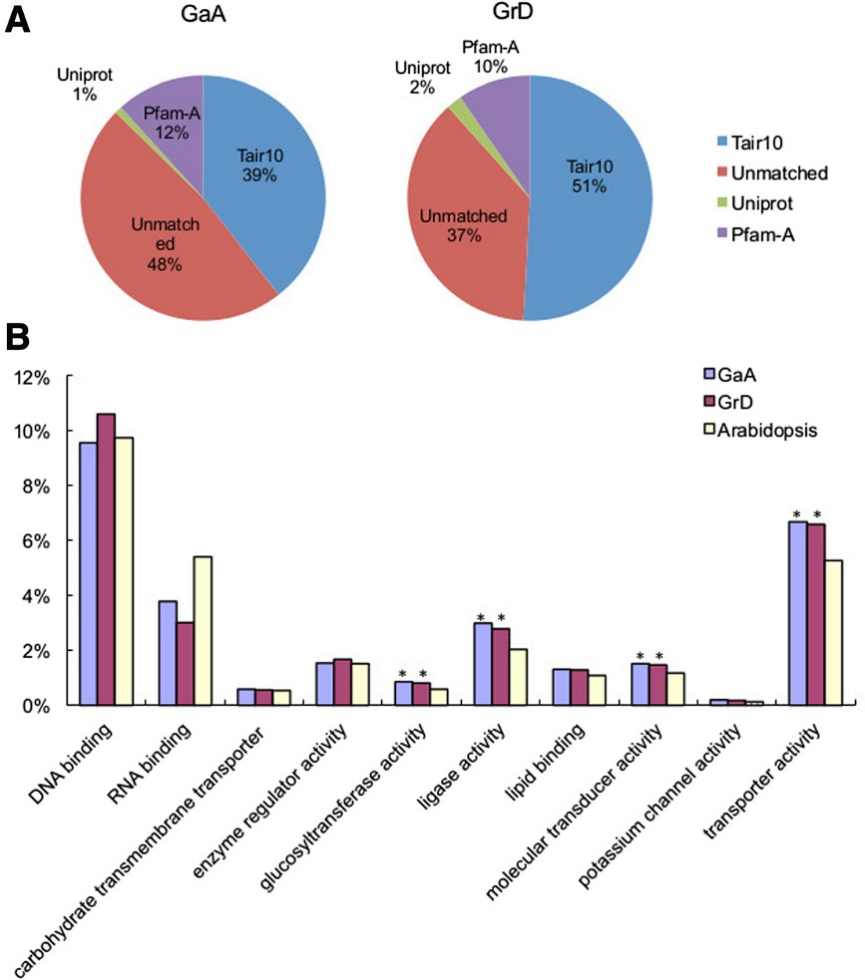
**Analysis of homologous transcripts against protein and peptide databases. (A)** Distribution of top BLASTX hits in GaA and GrD ESTs against known protein databases, TAIR10, Uniprot and Pfam-A, with a cut-off e-value of e^-10^. **(B)** Gene Ontology (GO) analysis using categories of molecular functions (* indicates GO categories that were enriched at the statistical significant level, p <0.05).

Proteins and peptides encoded by GaA and GrD ESTs were assigned using BLAST against multiple protein databases. With the e-value below e^-10^ and the identity of >50% of aligned amino-acid sequences, 39.4% of GaA contigs and 50.9% of GrD contigs matched targets in the *Arabidopsis* protein database (Figure 4A). A total of 35, 335 GaA contigs and 33, 333 GrD contigs matched 13,851 and 14,222 *Arabidopsis* protein-coding sequences, respectively. Additionally, the 11.7% of GaA contigs (10, 493) and 9.6% of GrD contigs (6, 300) matched protein entries in the Pfam-A database [27], using the e-value less than e-^05^. Additional 0.9% and 2% of GaA (846) and GrD (1, 328) contigs, respectively matched entries in the Uniprot database [28]. Approximately 48% of GaA contigs (46, 674) and 37% of GrD (40, 961) contigs could not be annotated with protein functions, which could be due to the genes related to cotton-specific biological processes such as fiber and cell wall biosynthesis, unknown transposable elements, non-coding RNAs, and/or computational errors.

### Gene ontology analysis of A and D ESTs

Gene ontology (GO) analysis using the molecular function classification showed significant increases of percentages of predicted genes in GaA and GrD ESTs relative to those in *Arabidopsis* genes in classes of glucosyltransferase, ligase, molecular transducer, and transporter activities (*P* < 1e^-4^, Fisher’s exact test) (Figure 4B). Cotton fiber cell differentiation and cell elongation require rapid carbohydrate biosynthesis and transport activities [29, 30]. The glucosyltransferase pathway is part of the primary metabolic process, which is required for biosynthesis of cellulose and primary and secondary cell walls. In addition, transport activities are required for rapid transport of sucrose and other sugars and nutrients that are important to fiber cell biosynthesis. Enrichment of the genes in these GO classes in cotton A and D-genome ESTs suggests evolutionary conservation of biological activities related to fiber production in cotton.

### Identification and characterization of genome-specific SNPs (GNPs)

**Figure 5:**
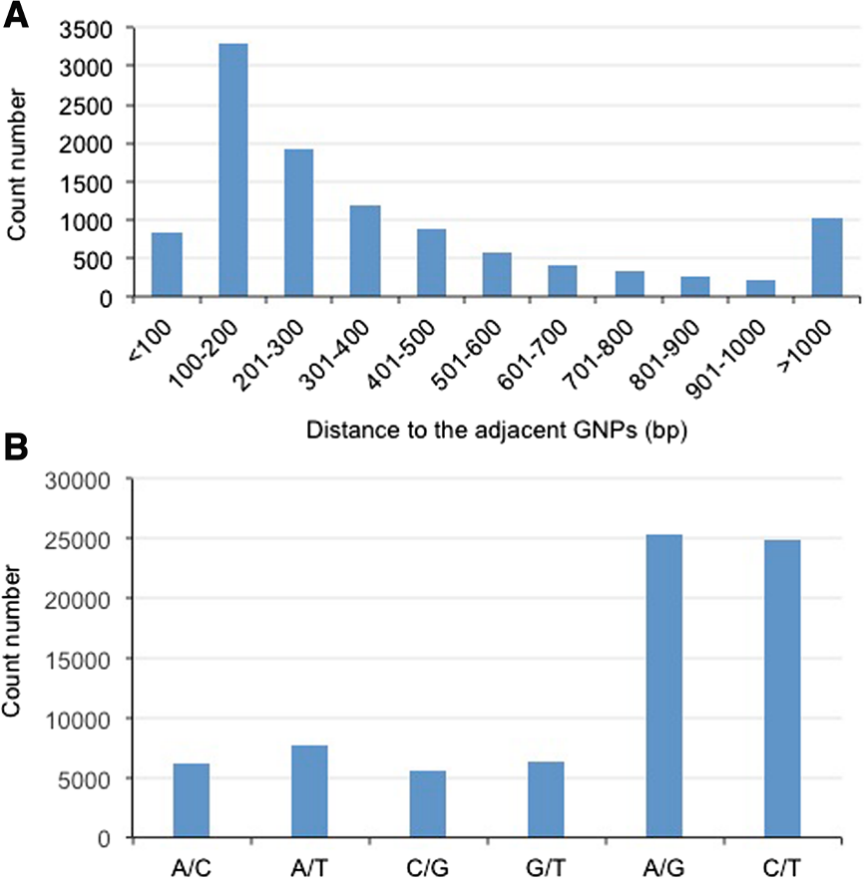
**Identification and characterization of Genome-specific single nucleotide polymorphism (GNPs) between diploid cotton GaA and GrD. (A)** Frequencies (Y-axis) of the distance to adjacent GNPs, which was not evenly distributed. Over 70% of GNPs were biased in a range from 100- to 600-bp. **(B)** Frequencies (Y-axis) of all possible types of transversion and transition (X-axis).

GNP is useful to discriminate allelic expression differences between species and in allotetraploid cotton. Here we analyzed and generated high-quality GNPs by the mapping A-genome 454 raw reads to D contigs using Newbler 2.3 (http://454.com/products/analysis-software/index.asp). The mapping efficiency is ~61%. Using high-stringent parameters (> = 90% identity, > = 8 × read coverage, and Q-value > =25), we identified 75,754 GNPs and 2,390 INDELs (insertion or deletion of bases) between A- and D-genome contigs (Additional file 2: DataSet1 and Additional file 3: DataSet2). More than 10,885 contigs, that represented 29% of the shared genes between A- and D-genomes, were aligned with at least one GNP. These GNP-containing contigs included genes encoding MYB transcription factors, epigenetic factors, and other protein factors important to fiber development (Additional file 4: Table S2). Cotton GNPs were not evenly distributed throughout expressed transcripts. The average length between GNPs is 300-bp, and most GNPs appeared on every 100–600 bp (Figure 5A).

GNPs may result from transition or transversion. Transition results from the interchanges within two-ring purines (A/G) or one-ring pyrimidines (C/T), while transversion is the interchange between two-ring purine and one-ring pyrimidine bases. Thus, transition is generally more frequent than transversion because the nature of these changes. The ratio between transition and transversion was 1.9:1 between A and D-genome ESTs) (Figure 5B), which is lower than the ratio (2.3:1) between the two allotetraploids, *G. hirsutum* and *G. barbadense*, as previously reported [31]. Both *G. hirsutum* and *G. barbadense* are evolved from the same ancestors of ancient A and D genome. Less transitions/transversions ratio between diploid cotton species than between allotetraploid species may reflect an evolutionary distance, which is larger in the former than in the latter.

Prediction of GNPs in GrD ESTs was computationally validated using the D-genome sequence [7]. Among 77,267 annotated transcripts in the D-genome, there were only 917 SNPs in 336 (0.4%) transcripts and 124 INDELs in 91 (0.1%) transcripts between annotated transcripts and GrD ESTs. This small amount of discrepancy suggests a low amount of sequence variation between these two sequencing libraries from the same *G. raimondii* accession likely due to a small population size.

To validate computationally predicted GNPs, we amplified 200–300-bp fragments that flank individual GNPs in *G. arboreum* (A-genome), *G. raimondii* (D-genome), and the allotetraploid *G. hirsutum* TM-1 (AD-genome) by PCR and sequenced individual fragments. Among 72 candidate GNPs for validation (Additional file 5: Figure S1), 52 (72.2%) perfectly matched the GNPs, which were found in the GaA and GrD ESTs were also present in A and D subgenomes in the allotetraploid. The remaining 20 (28.8%) GNPs showed biased amplifications towards one of the subgenomes in the allotetraploid. This could be due to non-specific amplification using the primer pairs in the PCR, sequence variation between the exact progenitors in the allotetraploid and extant diploid species used in the assay, and/or sequence changes between A and D-subgenomes after allotetraploid formation, as predicted in another study [17]. These data suggest that these EST assemblies in A- and D-genome species are useful resources for genome annotation and gene expression studies in allotetraploid cotton.

## Conclusions

We sequenced and assembled high-quality ESTs, including 89,588 contigs in *G. arboreum* (A-genome) and 65,542 contigs in *G. raimondii* (D-genome), which matched ~80% of EST collections in Cotton Gene Index 11 (CGI11, March 2012). About 29–38% of EST assemblies were unique in these two species. Sequence analysis showed that nearly 50% of ESTs were redundant in each progenitor species, together with the results of evolution rate analysis, suggesting that diploid cotton species underwent at least one round of whole genome duplication in their evolutionary history in a similar timeframe. Over 73% ESTs were shared between A and D-progenitor species, whereas over 20% or more ESTs were unique to each species. These ESTs were used to develop a list of 75,754 GNPs, which can be used to discriminate ~29% of homoeologous genes in allotetraploid cotton.

## Methods

### Plant material and RNA preparation

*G. arboreum* L*.* (A2) plants were grown in a greenhouse at 30 to 35°C under the natural light that is supplemented with fluorescent illumination for 16 hours (light) and 8 hours (dark) each day. The vegetative tissues included young roots, cotyledons, hypocotyls, true leaves, and stems from 4-week old seedlings after seed germination, Other tissues included ovules at -3 days post anthesis (DPA), 0, 3, 5, 10, and 15, and fibers at 3, 5, 10, and 15 DPA. Roots, shoots, leaves, and flowers of *G. raimondii* Ulbr. (D5) were provided by David M. Stelly’s lab at Texas A&M University. Plant tissues were grounded in liquid nitrogen and transferred to the lysis buffer that contained 2% CTAB, 2% polyvinylpyrrolidone (PVP) K-30 (soluble), 100 mM Tris–HCl (pH 8.0), 25 mM EDTA, 2.0 M NaCl, and 2% β-mercaptoethanol. The mixture was incubated at 65°C for 15 min. The solution containing RNA was extracted with chloroform:isoamyl alcohol (24:1) 3–5 times, and the final solution was added with 100% ethanol to precipitate RNA. The RNA pellet was briefly rinsed with 70% ethanol and dried in a vacuum centrifuge. RNA was dissolved in RNase-free water and quantified using NanoDrop (Thermo Scientific, Wilmington, DE).

### mRNA sequencing library preparation

Preparation for mRNA sequencing libraries followed the protocol of *de novo* transcriptome sequencing using 454-Titanium technology, which was developed by Mikhail V. Matz’s lab at The University of Texas at Austin [32]. The first stranded cDNA was normalized by duplex-specific nuclease in the Trimmer Kit from Evrogen (EA001, EA002, EA003). The libraries were sequenced by Titanium run on a Genome Sequencer FLX machine (Roche/454, Branford, CT) in the DNA Sequencing Center at Brigham Young University.

### Assemblies of 454 sequencing reads

The sequencing reads were assembled using Newbler 2.3 with the “runAssembly” command in the Lonestar supercomputer at the Texas Advanced Computing Center (TACC, Austin, TX). After the primary assembly, UCLUST with “usearch” command under the sequence identity option of 98% to remove the redundant transcripts. Two sets of sequencing reads were independently assembled and later merged using CAP3 with a quality score greater than 25, an overlap identity of 99%, an overlapping cutoff minimum of 50-bp, and other options as default. After the data were merged with 454 Titanium reads of A- and D-genome from Brigham Young Universtiy, the contigs fewer than 200-bp were purged from further analysis. EST assemblies in *G. arboreum* and *G. raimondii* were named GaA and GrD, respectively. Raw data of 454 sequencing have been deposited to the NCBI Sequence Read Archive (http://www.ncbi.nlm.nih.gov/sra/) under accession number SRX335546 and SRX335389.

### Ks distribution

Contig sequences were aligned to published *G. raimondii* protein sequences using blastx to detect open reading frames [33]. The predicted protein sequences of contigs were aligned for A vs A or D vs D to find paralogous genes of A- or D-genome using blastp with e-value less than e-10 [33]. For published *G. raimondii* genes, we identified paralogous genes through collinearity analysis using MCScanx and blastp (e-value ≤ e-10 ) [33, 34]. Paralogous genes were aligned using CLUSTALW under default parameters [35]. Then, we estimated Ks of paralogous genes by KaKs_Calculator using NG method [36].

### Gene ontology (GO) analysis

EST contigs in the A- and D-genome assemblies were searched against the *Arabidopsis* protein database (TAIR10, e-value < 1e^-10^) using BLASTx. GO classes of EST contigs were annotated using *Arabidopsis* homologous genes. The Fisher’s exact test was used to test if any GO class was significantly enriched in GaA and GrD assemblies when comparing with all genes in the *Arabidopsis* genome with a p-value of <1e^-4^.

### Identification of GNPs between GaA and GrD ESTs

To identify high-quality GNPs, we aligned 454 reads of A-genome onto GrD ESTs and genomic sequences using Newbler 2.3 with the “runMapping” command. A nucleotide-polymorphism was considered mapped if a high-quality match (99% identity or greater) was found across the flanking sequence of no less than 200-bp. The position with the polymorphism that matched more than 8 reads with identity of > 90% was counted to be a GNPs. Absence or presence of one or more nucleotides was considered as INDEL (deletion or insertion polymorphism).

### GNPs validation

Primer pairs were designed to amplify the fragment flanking the GNPs. The primer sequences were shown in Additional file 6: Table S3 and Additional file 7: Table S4. The PCR product size was predicted to be 200–300-bp. Genomic DNA of *G. arboreum, G. raimondii*, and *G. hirsutum* (TM-1) was used for PCR amplification. PCR products showing single fragments on a 4%-argrose gel were cleaned with the PCR Purification Kit (Qiagen, cat # 28106, Valencia, CA) and sequenced.

### Availability of supporting data

Raw data of 454 sequencing have been deposited to the NCBI Sequence Read Archive (http://www.ncbi.nlm.nih.gov/sra/) under accession number SRX335546 and SRX335389.

## Electronic supplementary material

Additional file 1: Table S1: Contig size and range of GaA and GrD after merging. (XLS 22 KB)

Additional file 2: DataSet1: Genomic Specific SNPs(GNPs) between GaA and GrD. (TXT 2 MB)

Additional file 3: DataSet2: Indel between GaA and GrD. (TXT 63 KB)

Additional file 4: Table S2: Selection of allele-separable genes. (XLS 28 KB)

Additional file 5: Figure S1: GNP validation. (A) The PCR amplicon with potential DNA polymorphysm from genomic DNA from *G. arboreum* (A2), *G. raimondii* (D5) and *G. hirsutum* (AADD). The code is according to the experiment # in **Table S3.** (B) Two examples for validated SNP sites in TM-1 genomic DNA. The arrow indicates the SNP site show single peak in diploid sequence and double peak in allotetraploid. (PDF 370 KB)

Additional file 6: Table S3: SNP validation candidates for functional-selected samples. (XLS 14 KB)

Additional file 7: Table S4: SNP validation candidates list for random-selected sample. (XLS 16 KB)

## Abbreviations

cDNA: Complementary deoxyribonucleic acid
GNPs: Genome-specific single nucleotide polymorphism
CGI11: Cotton Gene Index 11
EST: Expressed sequence tag
QTL: Quantative trait locus
INDEL: Insertion and deletion of bases on DNA.
