# Supplementary material for: Transcriptome analysis of extant cotton progenitors revealed tetraploidization and identified genome-specific single nucleotide polymorphism in diploid and allotetraploid cotton
Source: BMC Res Notes. 2014 Aug 6;7:493. doi: 10.1186/1756-0500-7-493 (PMC4267057; doi:10.1186/1756-0500-7-493)
Supplement: Supplementary file 5 — Additional file 5: Figure S1: GNP validation. (A) The PCR amplicon with potential DNA polymorphysm from genomic DNA from G. arboreum (A2), G. raimondii (D5) and G. hirsutum (AADD). The code is according to the experiment # in Table S3. (B) Two examples for validated SNP sites in TM-1 genomic DNA. The arrow indicates the SNP site show single peak in diploid sequence and double peak in allotetraploid. (PDF 370 KB) [file 13104_2014_3038_MOESM5_ESM.pdf]

Supplementary Figure 1

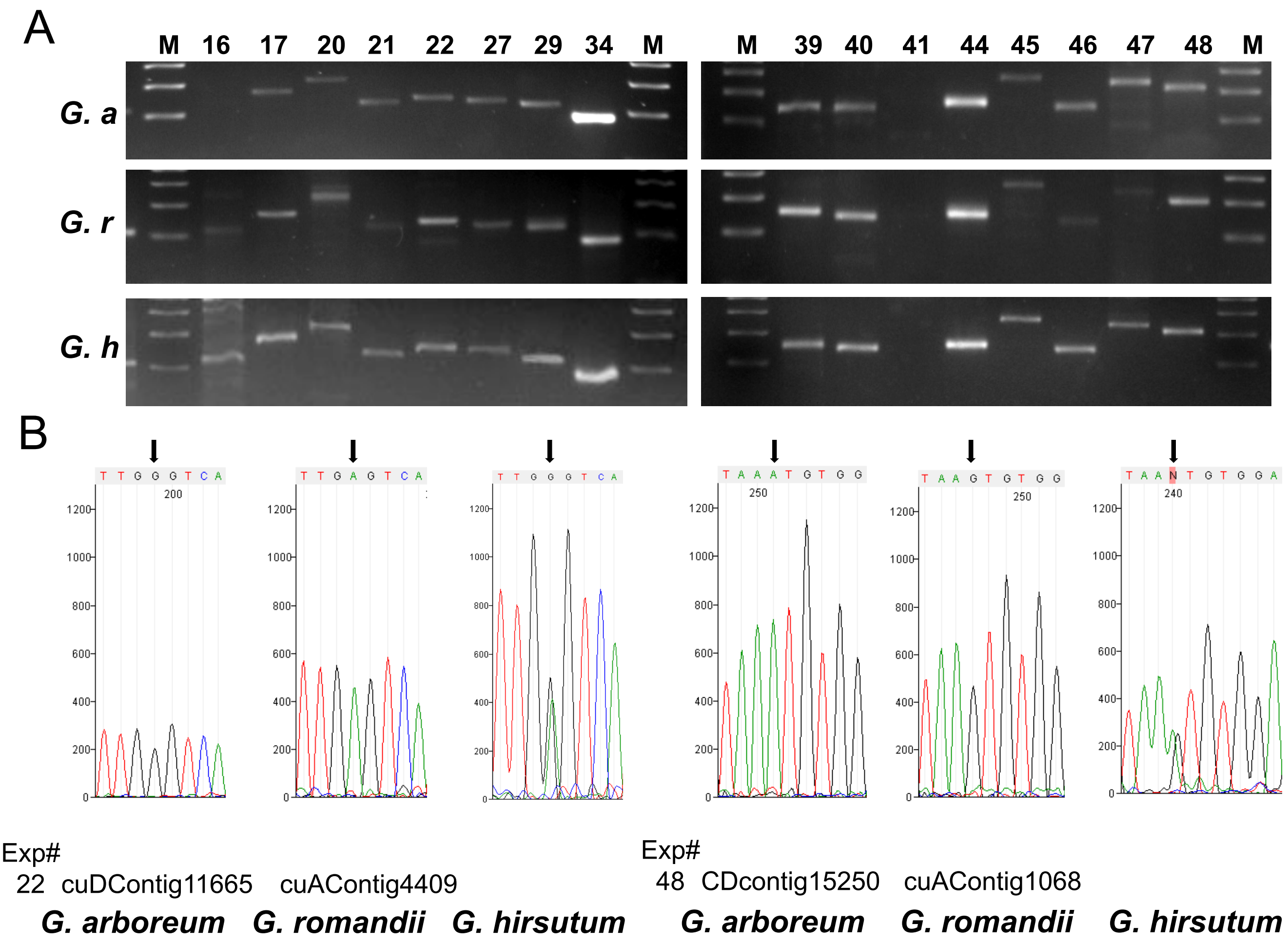

Supplementary Figure 1: GNP validation.
(**A**) The PCR amplicon with potential DNA polymorphysm from genomic DNA from *G. arboreum* (A2), *G. raimondii* (D5) and *G. hirsutum* (AADD). The code is according to the experiment # in table S3. (**B**) Two examples for validated SNP sites in TM-1 genomic DNA. The arrow indicates the SNP site show single peak in diploid sequence and double peak in allotetraploid.
